# Blood miRNAs Are Linked to Frequent Asthma Exacerbations in Childhood Asthma and Adult COPD

**DOI:** 10.3390/ncrna8020027

**Published:** 2022-04-03

**Authors:** Anshul Tiwari, Brian D. Hobbs, Jiang Li, Alvin T. Kho, Samir Amr, Juan C. Celedón, Scott T. Weiss, Craig P. Hersh, Kelan G. Tantisira, Michael J. McGeachie

**Affiliations:** 1 Channing Division of Network Medicine, Brigham and Women’s Hospital and Harvard Medical School, Boston, MA 02115, USA; reati@channing.harvard.edu (A.T.); rebdh@channing.harvard.edu (B.D.H.); cougarlj@163.com (J.L.); alvin_kho@hms.harvard.edu (A.T.K.); restw@channing.harvard.edu (S.T.W.); craig.hersh@channing.harvard.edu (C.P.H.); 2 Division of Pulmonary and Critical Care Medicine, Brigham and Women’s Hospital and Harvard Medical School, Boston, MA 02115, USA; 3 Computational Health Informatics Program, Boston Children’s Hospital, Boston, MA 02115, USA; 4 Translational Genomics Core, Mass General Brigham Personalized Medicine, Cambridge, MA 02139, USA; samr@bwh.harvard.edu; 5 Division of Pediatric Pulmonary Medicine, UPMC Children’s Hospital of Pittsburgh, University of Pittsburgh, Pittsburgh, PA 15224, USA; juan.celedon@chp.edu; 6 Division of Pediatric Respiratory Medicine, Rady Children’s Hospital, University of California, San Diego, CA 92123, USA; ktantisira@health.ucsd.edu

**Keywords:** asthma, COPD, miRNA, exacerbations, GACRS, differential expression, MAPK

## Abstract

MicroRNAs have been independently associated with asthma and COPD; however, it is unclear if microRNA associations will overlap when evaluating retrospective acute exacerbations. **Objective:** We hypothesized that peripheral blood microRNAs would be associated with retrospective acute asthma exacerbations in a pediatric asthma cohort and that such associations may also be relevant to acute COPD exacerbations. **Methods:** We conducted small-RNA sequencing on 374 whole-blood samples from children with asthma ages 6–14 years who participated in the Genetics of Asthma in Costa Rica Study (GACRS) and 450 current and former adult smokers with and without COPD who participated in the COPDGene study. **Measurements and Main Results:** After QC, we had 351 samples and 649 microRNAs for Differential Expression (DE) analysis between the frequent (*n* = 183) and no or infrequent exacerbation (*n* = 168) groups in GACRS. Fifteen upregulated miRs had odds ratios (OR) between 1.22 and 1.59 for a doubling of miR counts, while five downregulated miRs had ORs between 0.57 and 0.8. These were assessed for generalization in COPDGene, where three of the upregulated miRs (miR-532-3p, miR-296-5p, and miR-766-3p) and two of the downregulated miRs (miR-7-5p and miR-451b) replicated. Pathway enrichment analysis showed MAPK and PI3K-Akt signaling pathways were strongly enriched for target genes of DE miRNAs and miRNAs generalizing to COPD exacerbations, as well as infection response pathways to various pathogens. **Conclusion:** miRs (451b; 7-5p; 532-3p; 296-5p and 766-3p) associated with both childhood asthma and adult COPD exacerbations may play a vital role in airflow obstruction and exacerbations and point to shared genomic regulatory machinery underlying exacerbations in both diseases.

## 1. Introduction

Asthma is a disease characterized by airflow obstruction that is often, but not always, reversible. Asthma exacerbations, defined by hospitalizations, or emergency department visits for asthma, cause substantial healthcare costs and morbidity. Similarly, severe Chronic Obstructive Pulmonary Disease (COPD) exacerbations are costly [1] and accelerate the rate of irreversible pulmonary function decline [2]. While a genetic (or genomic) link between asthma and COPD was postulated in the Dutch Hypothesis [3], there has been modest genetic overlap found between asthma and COPD, and little work focused on shared omics determinants of exacerbations. Recent results show that looking to regulatory genomics, such as mRNA repression by microRNAs, can identify common genomic elements of asthma and COPD [4].

MicroRNAs (miRNAs) are small non-coding RNAs that regulate their target mRNAs post-transcriptionally through degradation or translational repression. Approximately 60% of mRNAs may be the targets of miRNAs [4,5]. miRNAs have been shown to regulate immune and inflammatory responses in various tissues, thus emerging as key molecules in asthma and COPD [6]. Previous studies of miRNAs in asthma focused mainly on asthma *per se*, including studies of circulating miRNA expression in children with asthma compared to healthy controls, regulation of IL-5 expression by miRNA differential expression in serum of asthmatics and healthy controls, and differential expression of miRNA in epithelial and airway cells [7,8,9,10]. Recent work on miRNA sequencing in induced sputum has identified miRs associated with asthma severity and with a history of exacerbations, and specific miRs associated with neutrophilic signaling pathways [11]. However, the role of peripheral whole-blood miRNAs (distinct from circulating, cell-free miRs in serum) in asthma exacerbations has not been previously investigated.

Asthma exacerbations are typically due to airway inflammation that results from viral infection, bacterial infection, or other environmental exposures, such as allergens. In children, asthma usually results from eosinophilic inflammation (“T2-high asthma”), but neutrophilic inflammation (“T2-low asthma”) occurs in some cases and can be more severe or difficult to treat. Systemic inflammation often coexists with airway inflammation, and some studies have shown genomic signatures of asthma exacerbations in mononuclear cells [12] or whole blood [13,14,15]. Moreover, whole-blood gene expression signatures have been associated with poor asthma control [16], a risk factor for exacerbations. As is the case in asthma, peripheral blood gene expression signatures have been demonstrated in COPD, where exacerbations mechanisms are related to neutrophils, eosinophils, macrophages, and Th1 CD4 lymphocytes [17,18]. Studying whole blood is therefore a natural place to look for shared regulatory mechanisms between eosinophilic and neutrophilic airway diseases. We hypothesized that whole-blood miRNA would be associated with immune and inflammatory responses predisposing for asthma exacerbations in the year prior to ascertainment and that these would at least partially replicate in COPD exacerbations.

## 2. Methods

### 2.1. Study Population

Subject recruitment and study procedures for the Genetics of Asthma in Costa Rica Study (GACRS) have been described in detail elsewhere [19,20]. In brief, the GACRS included 1165 Costa Rican children ages 6 to 14 years with asthma, who were recruited from February 2001 to July 2011. Asthma was defined as at least two respiratory symptoms (wheezing, cough, or dyspnea) or a history of asthma attacks in the previous year and a high probability of having at least six great-grandparents born in the Central Valley of Costa Rica, as determined by a genealogist based on the paternal and maternal last names of each of the child’s parents. Study participants completed a protocol including a questionnaire on respiratory and general health, slightly modified from the questionnaire used in the Collaborative Study on the Genetics of Asthma [21] and translated into Spanish. Spirometry was conducted with a Survey Tach Spirometer (Warren E. Collins, Braintree, MA, USA) following American Thoracic Society recommendations. The study was approved by the Institutional Review Boards of the Hospital Nacional de Niños (San José, Costa Rica) and Brigham and Women’s Hospital (Boston, MA, USA). The current work is covered by Brigham and Women’s Hospital IRB# 2017P001799.

### 2.2. Generalization Population

The COPDGene study (ClinicaTrials.gov—NCT00608764) has been described in detail previously [22]. In brief, COPDGene is a prospective study of current and former smokers with at least 10 pack-years of smoking, with and without spirometry-defined COPD. We used available peripheral whole blood collected from the COPDGene 5-year follow-up visit (conducted from 2012 to 2017) and performed small-RNA sequencing in 450 participants. Institutional review board (IRB) approval was obtained at each of the participating study centers (see details of COPDGene study centers in the Supplement) prior to study initiation. All participants provided written informed consent.

### 2.3. Primary Outcome

In the GACRS, an asthma exacerbation was defined as a visit to the emergency department or urgent care for asthma, or a hospitalization for asthma, in the previous year. Our primary outcome was having had at least three vs. less than three asthma exacerbations in the year prior to the study, a threshold based on patterns of care utilization. 

Self-reported retrospective exacerbations were determined in the COPDGene study by asking study participants at the five-year follow-up visit if they experienced a “flare-up of chest trouble” requiring treatment in the 12 months preceding the study questionnaire [23]. Additionally, we defined severe COPD exacerbations as any exacerbation in the previous 12 months requiring a visit to an emergency department or a hospitalization. Severe exacerbations were dichotomized according to whether study participants reported zero or at least one severe exacerbation in the previous 12 months, a threshold chosen to maximize power. We limited our exacerbations analysis to participants with a history of moderate to severe COPD, spirometrically defined as a forced expiratory volume in 1 s (FEV_1_) less than 80% of predicted and an FEV_1_-to-forced vital capacity (FEV1/FVC) ratio < 0.7. Prior work on exacerbations in COPDGene has discussed this in greater detail [23]. 

### 2.4. Sample Sequencing and Quality Control

We performed small RNA sequencing on all available whole-blood samples (*n* = 374) from GACRS and separately on 450 whole-blood samples from COPDGene at Mass General Brigham Personalized Medicine core. GACRS blood samples were acquired at the time of phenotype assessment, between 2001 and 2005, and were stored at -80 degrees C until sequencing began in 2019. Small RNA-seq libraries were prepared using the NEXTflex Small RNA-Seq Kit v3 (PerkinElmer’s, Waltham, MA, USA), which has a maximum input of 10 uL (10 ng to 250 ng). First, adapters were ligated to the 3′ and 5′ ends of the RNA. Reverse transcription was then carried out to generate cDNA from the ligated RNA. Following reverse transcription, cDNA yield was amplified by PCR, utilizing a distinct barcoded PCR primer for each sample. Finally, the PCR product entered size selection using magnetic beads for libraries in the range of 140–160 bp, resulting in the isolation of the indexed miRNA libraries. Libraries were then pooled and sequenced on an Illumina NextSeq 550 high output flow cell, with run length 75 bp single reads, to generate ~10 M reads per sample. We used blockers for hemolysis-associated miRNAs hsa-miR-486-5p, hsa-miR-92a, and hsa-miR-451a (PerkinElmer, Waltham, MA, USA), which were added to the input sample prior to library preparation [24]. The COMPSRA pipeline [25] and BCBio smallRNA-seq (https://github.com/bcbio/bcbio-nextgen, accessed on 12 September 2019) pipelines were employed for quality control (QC) of the RNA-seq data. miRNAs with less than five mapped reads in at least 50% of subjects were removed. We used the guided Principal Component Analysis (gPCA) [26] package for the identification of batch effects in GACRS. PCA of COPDGene samples showed an extreme outlier batch, which was removed from further analysis.

### 2.5. Identification of Differentially Expressed miRNAs

Differentially Expressed miRNAs (upregulated and downregulated miRNAs) between frequent and no or infrequent exacerbation conditions were identified using DESeq2 [27] version 1.30.0 (R version 4.0.3), which uses negative binomial regression, with a Benjamini–Hochberg false discovery rate (FDR) correction for multiple testing. A significance threshold of 10% FDR was used. The analysis was performed with adjustment for age, sex, use of inhaled corticosteroids (ICS) in the previous year, and sequencing batch. Logistic regression was used to obtain estimates of effect size (betas and Odds Ratios) for a doubling of miR counts. 

Top DE miRNAs were assessed for association with COPD exacerbations using DESeq2 and adjusted for age, sex, smoking history (current vs. former), pack-years of cigarette smoking, race, and sequencing batch.

Associations of miRNA levels with IgE was performed using linear regression, and miRNA associations with high or low eosinophil counts (more or less than 300 per microliter) were performed using logistic regression. Both were adjusted as stated above. Clinical and demographic features were compared using a Chi-square test for dichotomous variables and a *t*-test for continuous variables. 

### 2.6. Functional Annotation of Differentially Expressed miRNAs

Target mRNA transcripts were identified for 20 DE miR between frequent and no and infrequent asthma exacerbation and 5 replicated miR separately using the Micro T-CDS [28], TarBase [29], and Target Scan [30] databases using the default thresholds through multiMiR package version 1.12 [31] (Supplemental Figure S1A,B). The union of targets of each miR were used for Kyoto Encyclopedia of Genes and Genomes (KEGG) [32] pathway analyses through the clusterProfiler package version 3.18.1 [33]. We considered an adjusted *p*-value threshold of ≤0.05 and a gene count of 3 or more to indicate significant enrichment of targeted genes for a pathway.

The web-based platform miRNet 2.0 [34] was used for the construction of miRNA-target gene network and enrichment analysis for the 5 replicated miRNAs. miRNet uses the list of DE miRNAs and retrieves the predicted and validated putative gene targets from Tarbase-8.0 and miRTarBase-8.0 for network construction. The KEGG database with a hypergeometric test was used for functional enrichment, with an FDR threshold of 0.05 considered significant. The clusterProfiler package was used for enrichment analysis and dot plot.

For the protein–protein interaction (PPI) network we used putative targets of the 5 replicated miRNAs, which were built and visualized by using the STRING version 11.0 [35] online database and the Cystoscope v3.7.2 visualization tool [36]. 

## 3. Results

### 3.1. Cohort Characteristics

Of 1165 children with asthma from the GACRS, peripheral whole-blood samples were available for 365 children (31.33%). Of these, 351 GACRS participants (96%) had sufficient exacerbation data to be classified in to two groups: no or infrequent (<=2) exacerbations (*n* = 168); and frequent (>2) exacerbations (*n* = 183) (Table 1). 

Children with frequent exacerbations were younger (8.9 vs. 9.4 years) and weighed less (31.4 vs. 34.2 kg) than those with no or infrequent exacerbations, although there was no difference in height. Differences in BMI showed a trend toward higher exacerbations with lower BMI (*p* = 0.063), although this did not reach statistical significance. Children with frequent exacerbations were more likely to have used ICS in the previous year and had a statistically significant improvement in bronchodilator response (6.4% vs. 4.4%) but a lower FEV_1_/FVC pre- and post-bronchodilator response (85.6% vs. 87.5% and 88.2% vs. 89.9%, respectively) than those with no or infrequent exacerbations. 

Characteristics of subjects undergoing small RNA sequencing in COPDGene are shown in Table 2.

### 3.2. miRNA Sequencing

Small-RNA sequencing was completed on 365 samples from Costa Rica. On average, these resulted in 12.4 million total reads (+/−0.9 million) per sample. Of these 1.0 +/− 0.2 million were deemed poor quality by FRED score <20. Another 405 thousand +/− 420 thousand were dropped for other reasons (poor 3′ or 5′ end reads). Of the remaining high-quality reads, 8.3 million +/− 0.74 million per sample were mapped to one of 4694 human miRNAs in miRBase version 22 [37]. An average of 2.7 +/− 1.9 million reads per sample were unmapped to human miRNAs. Eight samples with less than 100 k mapped reads were considered failed and removed, as well as one sample with more than 70 M mapped reads.

Oligonucleotide blockers were used to reduce the number of hemolysis-associated miRNAs 486-5p, 92a-3p, and 451a; these miRNAs were removed from analysis. Even with efforts to reduce their prevalence, these miRNAs made up 10.6%, 3.0%, and 9.1% of mapped reads per sample. Finally, miRNAs with less than five reads in 50% of passed samples were dropped.

### 3.3. Identification of Differentially Expressed miRNAs

**Figure 1:**
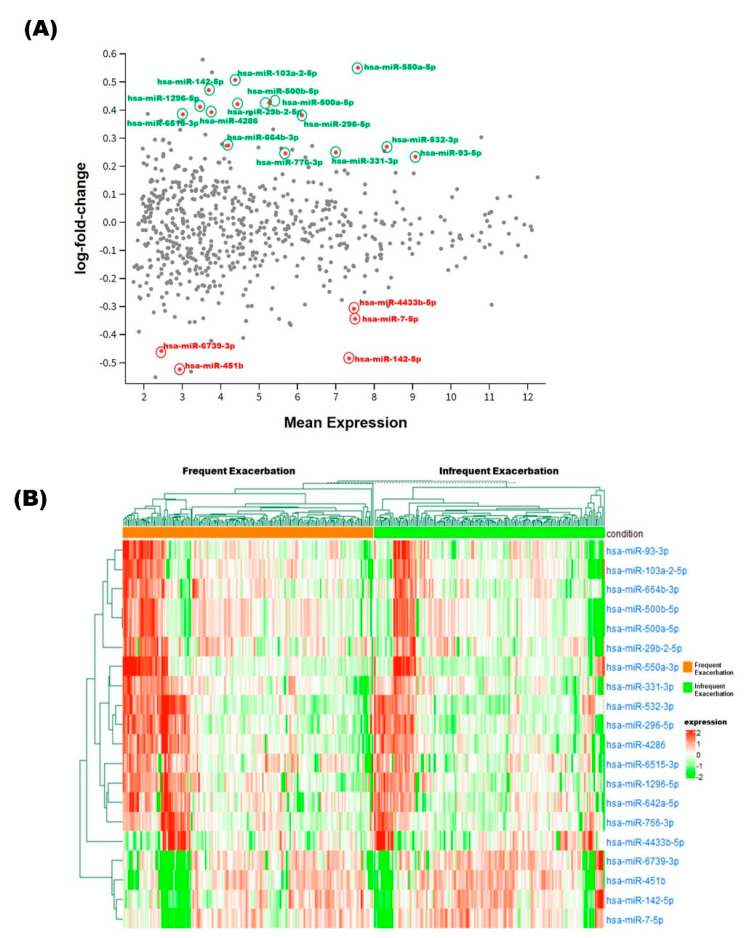
(**A**) Differential expression of miRNA between frequent and no or infrequent exacerbations in the GACRS. Green and red color circles indicate up and downregulated miRs, respectively. Mean expression shown in log2 scale. (**B**) Clustered heat map of all 20 differentially expressed miRs across conditions in the GACRS. DESeq-2 normalized expression counts (shifted logarithm transformation) were used. Unsupervised hierarchical clustering was used to generate the heat map and Pearson correlation was used as the distance metric.

After quality control, filtering, and normalization, we had 351 samples and 649 miRNAs for DE analysis between the groups with frequent (*n* = 183) and no or infrequent (*n* = 168) asthma exacerbations. We found 15 miRNAs showing higher expression and 5 miRNAs showing lower expression in subjects with frequent exacerbations (Table 3; Figure 1A). Effect estimates were computed and upregulated miRs had ORs between 1.22 and 1.59 for a doubling of miR counts, whereas downregulated miRs had ORs between 0.57 and 0.8 (Table 3). A clustered heat map of the 20 differentially expressed miRNAs is shown in Figure 1B. These 20 miRNAs were then tested for association with severe COPD exacerbations, in which miR-532-3p, miR-296-5p, and miR-766-3p were upregulated and miR-7-5p and miR-451b were downregulated (*p* < 0.05) in subjects with severe exacerbations (*n* = 24) compared to those without severe exacerbations (*n* = 122) in COPDGene (Table 4) (Supplemental Table S1). These five miRNAs therefore showed the same direction of effect with exacerbations in both the GACRS and COPDGene studies.

These five miRs were then tested for association with total Immunoglobulin E (IgE) and eosinophil counts (Table 5 and Table 6). Two miRs, miR-451b (*p* = 0.023), and miR-532-3p (*p* = 0.039) were significantly associated with IgE levels, while no miRs were significantly associated with eosinophils in GACRS. 

### 3.4. Identification of Putative Targets and Functional Assessment of Differentially Expressed miRNAs

**Figure 2:**
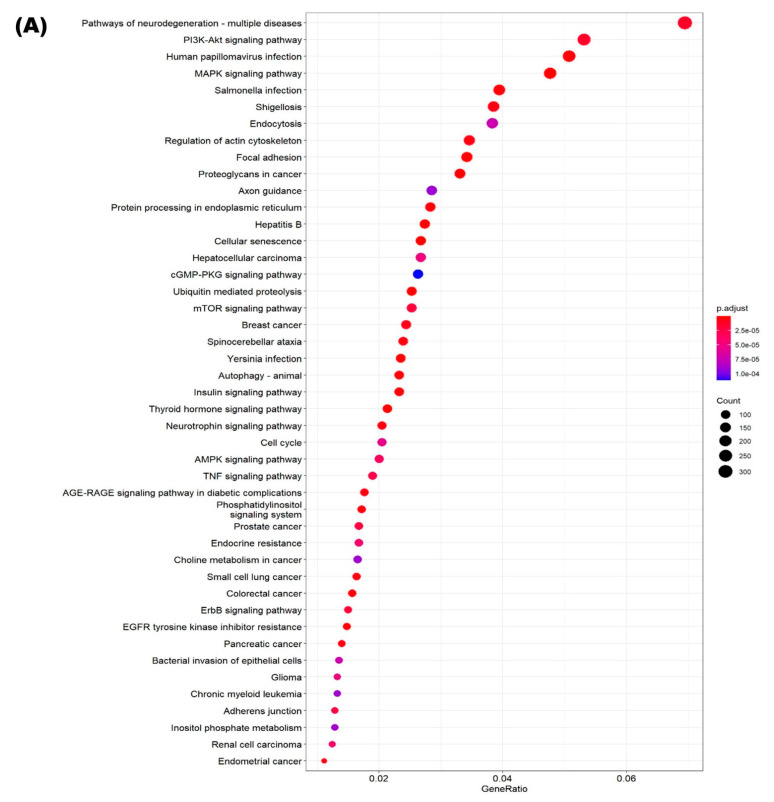

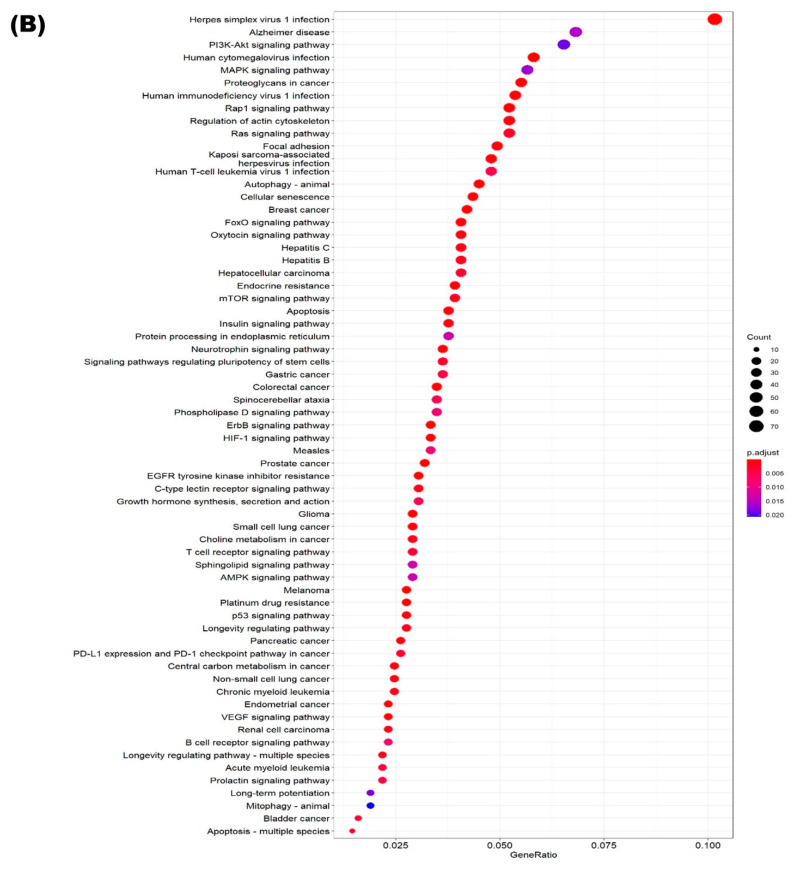
KEGG pathways enriched for DE miR target genes. (**A**) Pathways enriched for target genes of 20 DE miRs in the GACRS. Gene targets for miRs were identified using microT-CDS Diana, Target Scan & TarBase databases. (**B**) Pathways enriched for target genes from miRNet of 5 DE miRs generalizing to severe exacerbations in COPDGene.

**Figure 3:**
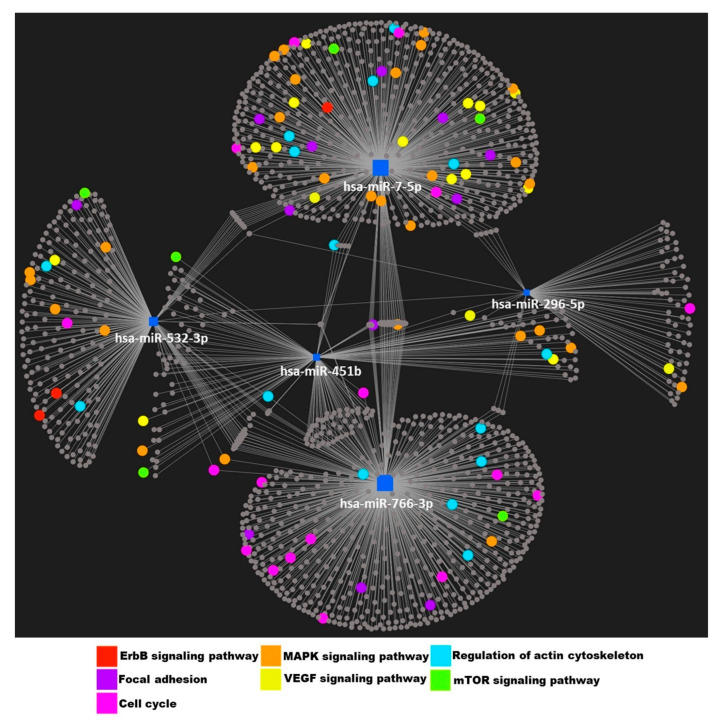
Network between 5 replicated miRs and gene targets. Nodes with different colors indicate genes in selected enriched KEGG pathways.

We performed an enrichment analysis of putative gene targets of the 20 DE miRNAs using the clusterProfiler package [33] (Figure 2A). Phosphatidylinositol 3-kinase (PI3K)—protein kinase B (Akt) and mitogen-activated protein kinase (MAPK) signaling pathways were among the top four most enriched pathways. We also separately considered the targets of only the five miRNAs that were also associated with severe exacerbations of COPD, where PI3K-Akt and MAPK signaling pathways were among the top five most enriched pathways (Figure 2B). The targets of these five miRNAs are shown in Figure 3, with highlighting for genes participating in enriched pathways previously associated with asthma. In general, this shows that the five miRNAs generalizing to severe COPD exacerbations show enrichment for established asthma pathways, with miR-7-5p mediating several signaling pathways and miR-766-3p mediating cell growth and morphology.

### 3.5. Protein–Protein Interaction Network

**Figure 4:**
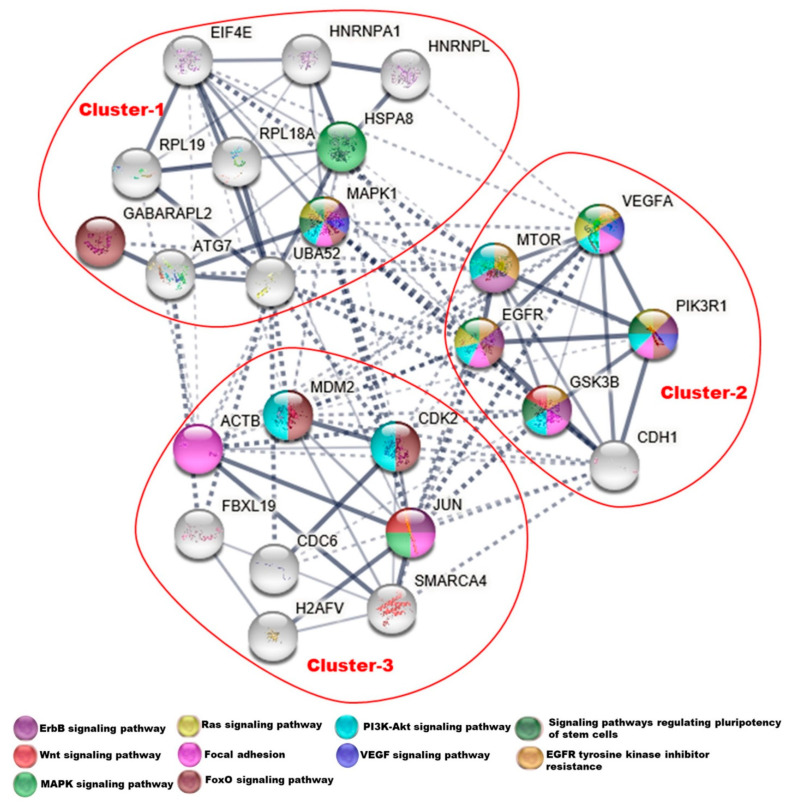
Protein–protein interaction network between hub proteins from targets of the 5 miRs generalizing to COPD exacerbations. Node coloration indicates membership in 10 significant asthma-related pathways. Dotted, light, and thick dark color line showing low, medium, and high edge confidence score, respectively.

From among the putative target genes of the 5 miRNAs generalizing to severe COPD exacerbations, 20 nodes with high degree were considered hub proteins: UBA52, EGFR, MAPK1, JUN, ACTB, VEGFA, CDH1, HSPA8, MDM2, MTOR, SMARCA4, GSK3B, PIK3R1, FBXL19, EIF4E, H2AFV, HNRNPA1, CDK2, ATG7, and RELA (Supplemental Table S2). Mapping these genes to the protein–protein interaction network (Figure 4) revealed three general clusters. KEGG pathway analysis of the genes in these modules showed that they were primarily enriched in the ErbB, RAS, PI3K-Akt, Wnt, vascular endothelial growth factor (VEGF), MAPK, FoxO signaling pathways, focal adhesion, epidermal growth factor receptor (EGFR) tyrosine kinase inhibitor resistance pathways and signaling pathways regulating pluripotency of stem cells. Cluster 2 was composed of hub genes with roles in a variety of asthma-related signaling pathways. Among these three clusters most of the cluster 2 genes showed the greatest enrichment for known asthma-related signaling pathways, followed by cluster 3 and then cluster 1. 

## 4. Discussion

We report that 20 miRNAs are significantly associated retrospectively with frequent asthma exacerbations in a study of Costa Rican children with asthma (the GACRS). Of these 20 miRNAs, 15 were upregulated and 5 were downregulated in subjects with frequent asthma exacerbations. Of these miRNAs, three (miR-532-3p, 296-5p, 766-3p) were upregulated and two were downregulated (7-5p and 451b) retrospectively in subjects with severe COPD exacerbations in the COPDGene study. miRs 451b and 532-3p were also associated with total IgE, a marker of inflammation and allergic response. These 20 miRs may be indicative of a general biological state that predisposes to more frequent exacerbations.

The five miRNAs that were differentially expressed between frequent and no or infrequent childhood asthma exacerbators, as well as between subjects with and without severe COPD exacerbations, have been previously associated with inflammation, though the weight of evidence varies for each miR. miR-296-5p has been associated with airway hyper-responsiveness in childhood asthma [38], and additionally linked to the MAPK, Wnt, and transforming growth factor beta (TGFB) signaling pathways, three key regulatory pathways in asthma pathogenesis [39]. Wnt [40] and TGFB [41] pathways have also been linked to COPD. miR-532-3p was shown to inhibit nuclear factor kappa-light-chain-enhancer of activated B cells (NF-kB) in vitro in response to corticosteroid treatment and was predictive of FEV_1_ gain on inhaled corticosteroid treatment in children with asthma [42]. In another study, miR-532-3p was part of an 11-miR signature that could distinguish ulcerative colitis from Crohn’s disease, providing evidence of its broad effect on inflammation. miR-766-3p has been associated with inflammation in rheumatoid arthritis, where this miR reduced NF-kB activation [43]. Of less relevance to asthma and COPD, miR-451b has been related to inflammation in lung cancer [44] and regulates cell proliferation, invasion, and apoptosis in many different conditions [45]. miR-7-5p was one of 55 miRNAs DE in an in vitro study of asthmatic airway epithelial cells, making it the miR with the least existing evidence linking it to asthma and COPD, although our network analysis showed gene targets of miR-7-5p to be enriched for a variety of asthma-related pathways (Figure 3). Our study adds substantially to this evidence by its large sample size and generalization to related exacerbation type.

Among children with asthma, Kho et al. [10] recently reported that 12 serum miRNAs were significantly associated with a milder type of asthma exacerbation (need for treatment with oral steroids) during one year of follow-up. Those 12 miRNAs are different from the 20 DE miRNAs in the current report. Those authors’ study design differs from ours in that they did miRNA profiling of serum from 160 children with asthma on inhaled corticosteroids from the United States and Canada using TaqMan arrays, while we used small-RNA sequencing in peripheral whole blood in 365 children from Costa Rica. Such differences likely account for the discrepant results between our two studies. 

Both gene targets of the 20 DE miRNAs and gene targets of the 5 miRNAs affecting both asthma and COPD exacerbations were enriched in MAPK PI3K-Akt and FoxO signaling pathways, which have been previously linked to asthma. MAPK signaling can contribute to both Th2-high eosinophilic asthma and Th2-low neutrophilic asthma, as well as COPD [46]. In Th2-high asthma, MAPK promotes Th2 cells to release IL-4, IL-5, and IL-13, stimulating IgE production, eosinophil recruitment, and airway hyperresponsiveness [47]. Cigarette smoke can trigger the MAPK pathway in small airway epithelial cells, causing a release of inflammatory cytokines and chemokines [48]. This type of activation can lead to airway remodeling in COPD and in Th2-low neutrophilic asthma, in turn leading to reduced lung function and greater risk of exacerbation [49]. In children with allergic asthma, Hu et al. (2020) discovered a link between indoleamine 2,3-dioxygenase (IDO) activity and Th17/regulatory T cells (Treg) imbalance. IDO may stimulate IL-10 synthesis while suppressing IL-6 expression, thus increasing Treg numbers. As a result, IDO could represent a molecular switch that leads to the conversion of Th17 cells to Tregs, thereby protecting against asthma etiology [50].

The PI3K-Akt pathway has a regulatory role in allergic asthma [51]. Activation of PI3K-Akt causes downstream activation of further signaling molecules, including NF-kB, itself a proinflammatory transcription factor. Inhibition of PI3K-Akt reduces expression of proinflammatory cytokines IL-4, IL-6, and IL-8, as well as Tumor Necrosis Factor alpha (TNF-a) and Immunoglobulin E (IgE); additionally, this pathway has been shown to be regulated by other miRNAs [52]. PI3K-Akt has also been implicated in inflammation in COPD and has been suggested as a possible therapeutic target in COPD [53]. The transcription factors fork-head box proteins O (FoxO) are involved in a variety of biological functions, including cell growth, metabolism, survival, and inflammation. FoxOs can serve as transcriptional activators and/or repressors when they are present in the nucleus and interact directly with DNA binding sites that have the FoxO consensus motif. The FoxO subclass in mammals is made up of four members: FoxO1, FoxO3, FoxO4, and FoxO6. Various chronic inflammatory diseases, such as rheumatoid arthritis and pulmonary hypertension, are linked to deregulation of FoxO1-mediated signalling in certain cell populations. FoxO signalling, presumably in the airways, is required to cope with very stressful situations, such as severe hypoxia. In addition, activated FoxO3A has been seen in airway epithelial cells of patients with COPD, cystic fibrosis, or ARDS (acute respiratory distress syndrome) pneumonia. In contrast, a different study discovered lower amounts of activated FoxO3A in COPD patients’ airway epithelial cells [54].

Apart from signalling pathways, PGD2 is a proinflammatory mediator produced by the cyclooxygenase-2 (COX-2) pathway and is derived from arachidonic acid. PGD2 is released during inflammatory reactions by activated immune cells, especially mast cells, and interacts with two receptors, PGD2 receptor 1 and 2 (DP1 and DP2), which can trigger thromboxane receptors at very low concentrations. DP2 is a G-protein-coupled receptor that is expressed on the membrane surface of Th2 cells, mast cells, and eosinophils. It is also known as the chemoattractant receptor homologous molecule expressed on Th2 cells (CRTH2). The binding of PGD2 to the DP2 receptor activates and migrates Th2 cells and eosinophils to the inflammatory sites in asthma, resulting in proinflammatory downstream signalling cascades [55]. 

The role of miRNAs in the control of epithelial pathobiology in asthma has been underlined in several recent studies. In vivo research in mice with OVA- or HDM-induced AAI, ex vivo/in vitro experiments involving luciferase reporter assays and stimulation–expression analysis, miRNA/mRNA microarrays, and in silico techniques were used by Bartel et al. (2017) [56]. The transcription factor cAMP-responsive element binding protein (Creb1) and its transcriptional coactivators (Crtc1-3) were identified as targets using this combined technique. They discovered that IL-13 treatment reduced the expression of Sec14-like 3 (Sec14l3), a possible Creb1 target, in both AAI models and primary normal human BECs, implying that miRNA-regulated Crtc1-3 and Sec14l3 play a role in early epithelial responses to type 2 stimuli. Extracellular vesicles (EVs) that transmit miRNAs across cells have recently been found as a unique intercellular communication method. It is worth noting that the extracellular miRNA pool in mice’s lungs was extremely comparable to that of the airway epithelium, with 80% of the discovered EVs being epithelial in origin [57].

Several of the top six or seven pathways enriched for targets of DE and generalizing miRNAs are responses to infection; while none of these specific pathways are related to respiratory infection, respiratory infections are a risk factor for asthma development and a proximate cause of asthma and COPD exacerbations. It may be that miRNAs regulating response to infection are key in asthma and COPD exacerbations, which are frequently caused by infections [58], particularly if dysregulation in these miRs establishes an in vivo environment predisposing to exacerbation.

We recognize our study has several limitations. We attempted to generalize our asthma exacerbation microRNA associations to individuals with COPD, for which asthma is a strong risk factor; however, the pathobiology of asthma and COPD exacerbations are not wholly overlapping, with both including substantial environmental and socio-economic risk factors. Five of twenty asthma exacerbation miRNAs showed generalization to severe COPD exacerbations, a separate but related phenotype, in a cohort of adult current and former smokers with moderate to severe COPD. Differences in cohort demographics, environmental exposures, and the pathology of asthma vs. COPD exacerbations may have predisposed to the null result of no miRNAs generalizing across conditions. Our analysis was cross-sectional and retrospective, so we cannot establish a firm temporal relationship between exacerbations and associated miRNAs; at best we could hypothesize these miRNAs lead to moderate systemic changes which may predispose to greater exacerbation frequency. One of the miRNAs, 451b, is structurally similar to a hemolysis-associated miR, which was blocked with an oligonucleotide. Although other studies have reported off-target effects of blockers on miR-451b [59], our own investigation has shown that to be unlikely with the particular blocker we used [24]. Asthma was characterized clinically in the GACRS cohort, which may have led to some misreporting or misdiagnosis.

## 5. Conclusions

20 DE miRNAs were associated with frequent asthma exacerbation in children. Five miRNAs, miR-532-3p, 296-5p, 766-3p, 7-5p and 451b, generalized to severe COPD exacerbations. Targets of these miRNAs were significantly enriched in a number of known asthma- and COPD-related pathways, including PI3-AKT and MAPK signaling. These results point to possible shared genomic regulatory mechanisms partly underlying exacerbations in these two diseases. 

## Figures and Tables

**Table 1:** Baseline Epidemiologic and Clinical Characteristics of the GACRS.

	Non-Exacerbation (*N* = 168)	Exacerbation (*N* = 183)	*p*-Value
** Gender **			
Male	73 (43.5%)	69 (37.7%)	0.324
Female	95 (56.5%)	114 (62.3%)	
** Age (years) **			
Mean (SD)	9.40 (1.83)	8.89 (1.88)	0.0109
Median [Min, Max]	9.29 [4.50, 13.3]	8.65 [0.271, 13.0]	
** Height (cm) **			
Mean (SD)	133 (15.7)	131 (11.7)	0.161
Median [Min, Max]	133 [0, 163]	129 [108, 167]	
**Weight (kg)**			
Mean (SD)	34.2 (12.2)	31.4 (11.1)	0.0245
Median [Min, Max]	30.9 [15.0, 71.7]	27.9 [17.0, 81.6]	
** BMI **			
Mean (SD)	18.6 (4.35)	17.8 (3.56)	0.0634
Median [Min, Max]	17.2 [11.3, 41.4]	16.8 [12.7, 34.0]	
** % Predicted Pre-BD FEV1 **			
Mean (SD)	98.6 (15.1)	100 (17.1)	0.423
Median [Min, Max]	97.5 [53.1, 144]	99.6 [46.2, 180]	
** % Predicted Pre-BD FVC **			
Mean (SD)	101 (15.3)	104 (16.0)	0.0662
Median [Min, Max]	99.0 [52.5, 151]	102 [54.6, 174]	
** FEV1/FVC pre-bronchodilator **			
Mean (SD)	87.5 (7.60)	85.6 (7.99)	0.0283
Median [Min, Max]	87.5 [65.3,100]	86.0 [61.8,99.9]	
** FEV1/FVC post-bronchodilator **			
Mean (SD)	89.9 (6.02)	88.2 (7.07)	0.0179
Median [Min, Max]	89.4 [67.0,100]	89.2 [66.4,100]	
** Bronchodilator Response as % of baseline FEV1 **			
Mean (SD)	4.38 (8.47)	6.37 (9.57)	0.0405
Median [Min, Max]	2.88 [−15.3,48.6]	5.10 [−16.3,47.2]	
** Inhaled Steroids **			
No	88 (52.4%)	70 (38.3%)	0.0108
Yes	80 (47.6%)	113 (61.7%)	
** Total IgE **			
Mean (SD)	1.79 (0.408)	1.81 (0.391)	0.596
Median [Min, Max]	2.00 [1.00,2.00]	2.00 [1.00,2.00]	
** Eosinophil Count **			
Mean (SD)	1.40 (0.491)	1.51 (0.501)	0.0444
Median [Min, Max]	1.00 [1.00,2.00]	2.00 [1.00,2.00]	

% predicted Pre-BD FEV1: Percent predicted pre-bronchodilator forced expiratory volume in one second. % predicted Pre-BD FVC: Percent predicted pre-bronchodilator forced vital capacity. % predicted FEV1/FVC post-BD: Percent predicted post-bronchodilator FEV1/FVC ratio. BD Response: Bronchodilator response as a percentage of pre-bronchodilator FEV1. Inhaled steroids: respondents indicating use of inhaled corticosteroids in previous year.

**Table 2:** Baseline Epidemiologic and Clinical Characteristics of COPDGene.

	No Exacerbations (*N* = 122)	Severe Exacerbations (*N* = 24)	*p*-Value
**Gender**			
Male	54 (44.3%)	10 (42.7%)	0.99
Female	68 (55.7%)	14 (58.3%)	
**Age (years)**			
Mean (SD)	68.6 (8.8)	69.5 (9.16)	0.64
**Race (% African American)**	27 (22.1)	7 (29.2)	0.63
**Current Smoking (%)**	46 (37.7)	8 (33.3)	0.86
**Pack Years Smoking**			
Mean (SD)	51.7 (26.6)	51.9 (28.8)	0.97
**% predicted FEV1**			
Mean (SD)	53.9 (16.5)	53.2 (17.6)	0.85
**FEV1/FVC**			
Mean (SD)	0.52 (0.12)	0.52 (0.11)	0.95
**Asthma Diagnosed before Age 40 (%)**	15 (12.3)	5 (20.8)	0.26

% predicted FEV1: Percent predicted forced expiratory volume in one second.

**Table 3:** Significant up and downregulated miRNAs between no or infrequent exacerbators and frequent exacerbators in the GACRS. Base Mean: normalized mean counts in reference group. Log2FC: base-2 fold change from no or infrequent to frequent exacerbators. *p*-value: computed with DESeq2. Beta and Odds Ratio from logistic regression are for a doubling of miR counts.

	Base Mean	log2FC	Beta	Odds Ratio	*p*-Value	FDR
hsa-miR-451b	18.29	−0.524	−0.065	0.57	1.88 × 10^−4^	2.65 × 10^−2^
hsa-miR-142-5p	1550.70	−0.485	−0.057	0.62	5.76× 10^−4^	4.15× 10^−2^
hsa-miR-6739-3p	11.14	−0.458	−0.063	0.64	8.29× 10^−4^	4.50× 10^−2^
hsa-miR-7-5p	1811.60	−0.344	−0.041	0.80	3.02× 10^−3^	9.80× 10^−2^
hsa-miR-4433b-5p	1759.78	−0.308	−0.059	0.80	2.36× 10^−3^	9.00× 10^−2^
hsa-miR-93-3p	8737.45	0.234	0.105	1.22	2.32×10^−3^	9.00× 10^−2^
hsa-miR-766-3p	294.18	0.246	0.065	1.31	2.80× 10^−3^	9.56× 10^−2^
hsa-miR-331-3p	1098.62	0.249	0.052	1.19	2.64× 10^−3^	9.53× 10^−2^
hsa-miR-532-3p	4142.15	0.27	0.102	1.25	9.33× 10^−4^	4.50× 10^−2^
hsa-miR-664b-3p	66.08	0.274	0.074	1.27	1.04× 10^−3^	4.50× 10^−2^
hsa-miR-296-5p	452.86	0.381	0.063	1.35	9.60× 10^−4^	4.50× 10^−2^
hsa-miR-6515-3p	19.91	0.385	0.064	1.43	4.79× 10^−4^	4.15× 10^−2^
hsa-miR-4286	42.35	0.392	0.049	1.37	9.82× 10^−4^	4.50× 10^−2^
hsa-miR-1296-5p	31.37	0.411	0.038	1.31	9.00× 10^−4^	4.50× 10^−2^
hsa-miR-29b-2-5p	84.16	0.421	0.081	1.48	5.79× 10^−5^	2.65× 10^−2^
hsa-miR-500b-5p	193.56	0.425	0.072	1.59	1.48× 10^−4^	2.65× 10^−2^
hsa-miR-500a-5p	197.54	0.431	0.072	1.59	1.20× 10^−4^	2.65× 10^−2^
hsa-miR-642a-5p	39.54	0.471	0.045	1.44	5.20× 10^−4^	4.15× 10^−2^
hsa-miR-103a-2-5p	79.21	0.507	0.043	1.50	2.04× 10^−4^	2.65× 10^−2^
hsa-miR-550a-3p	1953.34	0.55	0.045	1.40	3.17× 10^−4^	3.43× 10^−2^

**Table 4:** List of replicated up and downregulated miRNAs without exacerbations and those experiencing severe exacerbators in COPDGene.

	log2FC	*p*-Value
hsa-miR-451b	−0.636	0.054
hsa-miR-7-5p	−0.524	0.064
hsa-miR-532-3p	0.311	0.02
hsa-miR-296-5p	0.391	0.021
hsa-miR-766-3p	0.289	0.036

**Table 5:** List of replicated up and downregulated miRNAs and association with total IgE. *p*-value: computed with DESeq2. Beta and Odds Ratio (OR) from logistic regression are for a doubling of miR counts. 2.50%: lower bound of 95% confidence interval of odds ratio. 97.50%: upper bound of 95% confidence interval of odds ratio.

	*p*-Value	Beta	OR	2.50%	97.50%
hsa-miR-7-5p	0.094	0.031	0.031	−0.005	0.068
hsa-miR-451b	0.023	0.033	0.033	0.004	0.062
hsa-miR-296-5p	0.909	0.002	0.002	−0.036	0.041
hsa-miR-532-3p	0.039	−0.060	−0.060	−0.118	−0.003
hsa-miR-766-3p	0.962	0.001	0.001	−0.041	0.043

**Table 6:** List of replicated up and downregulated miRNAs and association with total Eosinophil count. *p*-value: computed with DESeq2. Beta and Odds Ratio (OR) from logistic regression are for a doubling of miR counts. 2.50%: lower bound of 95% confidence interval of odds ratio. 97.50%: upper bound of 95% confidence interval of odds ratio.

	*p*-Value	Beta	OR	2.50%	97.50%
hsa-miR-7-5p	0.325	0.023	0.023	−0.023	0.071
hsa-miR-451b	0.904	0.002	0.002	−0.034	0.039
hsa-miR-296-5p	0.516	0.016	0.016	−0.033	0.066
hsa-miR-532-3p	0.449	−0.028	−0.028	−0.103	0.045
hsa-miR-766-3p	0.183	0.036	0.036	−0.017	0.090

## Data Availability

Primary miRNA data is in submission to GEO. GEO Accession number pending.

## Supplementary Materials

The following are available online at https://www.mdpi.com/article/10.3390/ncrna8020027/s1, Figure S1: Number of putative targets retrieved from microT-CDS, TarBase and TargetScan (A) for 20 DE miRNAs. (B) 5 replicated miRNAs frequent and infrequent severe exacerbations; Table S1: t List of all up- and down-regulated miRNAs between subjects without exacerbations and those experiencing severe exacerbators in COPDGene; Table S2: List of 20 hub proteins from STRING; COPDGene Phase 3.

## References

[1] May S.M., Li J.T. (2015). Burden of chronic obstructive pulmonary disease: Healthcare costs and beyond. Allergy Asthma Proc..

[2] Donaldson G.C., Seemungal T.A., Bhowmik A., Wedzicha J.A. (2002). Relationship between exacerbation frequency and lung function decline in chronic obstructive pulmonary disease. Thorax.

[3] Postma D.S., Weiss S.T., van den Berge M., Kerstjens H.A., Koppelman G.H. (2015). Revisiting the Dutch hypothesis. J. Allergy Clin. Immunol..

[4] Tiwari A., Li J., Kho A.T., Sun M., Lu Q., Weiss S.T., Tantisira K.G., McGeachie M.J. (2021). COPD-associated miR-145-5p is downregulated in early-decline FEV1 trajectories in childhood asthma. J. Allergy Clin. Immunol..

[5] O’Brien J., Hayder H., Zayed Y., Peng C. (2018). Overview of MicroRNA Biogenesis, Mechanisms of Actions, and Circulation. Front. Endocrinol..

[6] De Smet E.G., Mestdagh P., Vandesompele J., Brusselle G.G., Bracke K.R. (2015). Non-coding RNAs in the pathogenesis of COPD. Thorax.

[7] Chandan K., Gupta M., Sarwat M. (2019). Role of Host and Pathogen-Derived MicroRNAs in Immune Regulation During Infectious and Inflammatory Diseases. Front. Immunol..

[8] Rupani H., Sanchez-Elsner T., Howarth P. (2013). MicroRNAs and respiratory diseases. Eur. Respir. J..

[9] Tahamtan A., Teymoori-Rad M., Nakstad B., Salimi V. (2018). Anti-Inflammatory MicroRNAs and Their Potential for Inflammatory Diseases Treatment. Front. Immunol..

[10] Kho A.T., McGeachie M.J., Moore K.G., Sylvia J.M., Weiss S.T., Tantisira K.G. (2018). Circulating microRNAs and prediction of asthma exacerbation in childhood asthma. Respir. Res..

[11] Gomez J.L., Chen A., Diaz M.P., Zirn N., Gupta A., Britto C., Sauler M., Yan X., Stewart E., Santerian K. (2020). A Network of Sputum MicroRNAs Is Associated with Neutrophilic Airway Inflammation in Asthma. Am. J. Respir. Crit Care Med..

[12] Bjornsdottir U.S., Holgate S.T., Reddy P.S., Hill A.A., McKee C.M., Csimma C.I., Weaver A.A., Legault H.M., Small C.G., Ramsey R.C. (2011). Pathways activated during human asthma exacerbation as revealed by gene expression patterns in blood. PLoS ONE.

[13] Tsalik E.L., Henao R., Nichols M., Burke T., Ko E.R., McClain M.T., Hudson L.L., Mazur A., Freeman D.H., Veldman T. (2016). Host gene expression classifiers diagnose acute respiratory illness etiology. Sci. Transl. Med..

[14] Lydon E.C., Bullard C., Aydin M., Better O.M., Mazur A., Nicholson B.P., Ko E.R., McClain M.T., Ginsburg G.S., Woods C.W. (2019). A host gene expression approach for identifying triggers of asthma exacerbations. PLoS ONE.

[15] Gomez J.L., Diaz M.P., Nino G., Britto C.J. (2018). Impaired type I interferon regulation in the blood transcriptome of recurrent asthma exacerbations. BMC Med. Genom..

[16] Croteau-Chonka D.C., Qiu W., Martinez F.D., Strunk R.C., Lemanske R.F., Liu A.H., Gilliland F.D., Millstein J., Gauderman W.J., Ober C. (2017). Gene Expression Profiling in Blood Provides Reproducible Molecular Insights into Asthma Control. Am. J. Respir. Crit. Care Med..

[17] Barnes P.J. (2008). Immunology of asthma and chronic obstructive pulmonary disease. Nat. Rev. Immunol..

[18] Morrow J.D., Qiu W., Chhabra D., Rennard S.I., Belloni P., Belousov A., Pillai S.G., Hersh C.P. (2015). Identifying a gene expression signature of frequent COPD exacerbations in peripheral blood using network methods. BMC Med. Genom..

[19] Kho A.T., Sordillo J., Wu A.C., Cho M.H., Sharma S., Tiwari A., Lasky-Su J., Weiss S.T., Tantisira K.G., McGeachie M.J. (2020). CASTER: Cross-Sectional Asthma STEroid Response Measurement. J. Pers. Med..

[20] Hunninghake G.M., Soto-Quiros M.E., Avila L., Ly N.P., Liang C., Sylvia J.S., Klanderman B.J., Silverman E.K., Celedon J.C. (2007). Sensitization to Ascaris lumbricoides and severity of childhood asthma in Costa Rica. J. Allergy Clin. Immunol..

[21] Blumenthal M.N., Banks-Schlegel S., Bleecker E.R., Marsh D.G., Ober C. (1995). Collaborative studies on the genetics of asthma--National Heart, Lung and Blood Institute. Clin. Exp. Allergy.

[22] Regan E.A., Hokanson J.E., Murphy J.R., Make B., Lynch D.A., Beaty T.H., Curran-Everett D., Silverman E.K., Crapo J.D. (2010). Genetic epidemiology of COPD (COPDGene) study design. COPD J. Chronic Obstr. Pulm. Dis..

[23] Han M.K., Kazerooni E.A., Lynch D.A., Liu L.X., Murray S., Curtis J.L., Criner G.J., Kim V., Bowler R.P., Hanania N.A. (2011). Chronic obstructive pulmonary disease exacerbations in the COPDGene study: Associated radiologic phenotypes. Radiology.

[24] LaBelle J., Bowser M., Brown A., Farnam L., Kho A., Li J., McGeachie M., Chase R., Piehl S., Allen K. (2021). Commercially Available Blocking Oligonucleotides Effectively Suppress Unwanted Hemolysis Related miRNAs in a Large Whole Blood RNA Cohort. J. Mol. Diagn..

[25] Li J., Kho A.T., Chase R.P., Pantano L., Farnam L., Amr S.S., Tantisira K.G. (2020). COMPSRA: A COMprehensive Platform for Small RNA-Seq data Analysis. Sci. Rep..

[26] Reese S.E., Archer K.J., Therneau T.M., Atkinson E.J., Vachon C.M., de Andrade M., Kocher J.P., Eckel-Passow J.E. (2013). A new statistic for identifying batch effects in high-throughput genomic data that uses guided principal component analysis. Bioinformatics.

[27] Love M.I., Huber W., Anders S. (2014). Moderated estimation of fold change and dispersion for RNA-seq data with DESeq2. Genome Biol..

[28] Paraskevopoulou M.D., Georgakilas G., Kostoulas N., Vlachos I.S., Vergoulis T., Reczko M., Filippidis C., Dalamagas T., Hatzigeorgiou A.G. (2013). DIANA-microT web server v5.0: Service integration into miRNA functional analysis workflows. Nucleic Acids Res..

[29] Karagkouni D., Paraskevopoulou M.D., Chatzopoulos S., Vlachos I.S., Tastsoglou S., Kanellos I., Papadimitriou D., Kavakiotis I., Maniou S., Skoufos G. (2018). DIANA-TarBase v8: A decade-long collection of experimentally supported miRNA-gene interactions. Nucleic Acids Res..

[30] Agarwal V., Bell G.W., Nam J.W., Bartel D.P. (2015). Predicting effective microRNA target sites in mammalian mRNAs. Elife.

[31] Ru Y., Kechris K.J., Tabakoff B., Hoffman P., Radcliffe R.A., Bowler R., Mahaffey S., Rossi S., Calin G.A., Bemis L. (2014). The multiMiR R package and database: Integration of microRNA-target interactions along with their disease and drug associations. Nucleic Acids Res..

[32] Kanehisa M., Sato Y., Kawashima M., Furumichi M., Tanabe M. (2016). KEGG as a reference resource for gene and protein annotation. Nucleic Acids Res..

[33] Yu G., Wang L.G., Han Y., He Q.Y. (2012). clusterProfiler: An R package for comparing biological themes among gene clusters. Omics J. Integr. Biol..

[34] Chang L., Zhou G., Soufan O., Xia J. (2020). miRNet 2.0: Network-based visual analytics for miRNA functional analysis and systems biology. Nucleic Acids Res..

[35] Szklarczyk D., Gable A.L., Lyon D., Junge A., Wyder S., Huerta-Cepas J., Simonovic M., Doncheva N.T., Morris J.H., Bork P. (2019). STRING v11: Protein-protein association networks with increased coverage, supporting functional discovery in genome-wide experimental datasets. Nucleic Acids Res..

[36] Shannon P., Markiel A., Ozier O., Baliga N.S., Wang J.T., Ramage D., Amin N., Schwikowski B., Ideker T. (2003). Cytoscape: A software environment for integrated models of biomolecular interaction networks. Genome Res..

[37] Kozomara A., Birgaoanu M., Griffiths-Jones S. (2019). miRBase: From microRNA sequences to function. Nucleic Acids Res..

[38] Davis J.S., Sun M., Kho A.T., Moore K.G., Sylvia J.M., Weiss S.T., Lu Q., Tantisira K.G. (2017). Circulating microRNAs and association with methacholine PC20 in the Childhood Asthma Management Program (CAMP) cohort. PLoS ONE.

[39] Zhang Y.H., Yang Y., Zhang C., Sun Y.F., Zhu W., Ma C.L., Zhou X.Y. (2016). [Prediction of microRNA-296-5p target genes and its application in lung development]. Zhongguo Dang Dai Er Ke Za Zhi.

[40] Skronska-Wasek W., Mutze K., Baarsma H.A., Bracke K.R., Alsafadi H.N., Lehmann M., Costa R., Stornaiuolo M., Novellino E., Brusselle G.G. (2017). Reduced Frizzled Receptor 4 Expression Prevents WNT/beta-Catenin-driven Alveolar Lung Repair in Chronic Obstructive Pulmonary Disease. Am. J. Respir. Crit. Care Med..

[41] Verhamme F.M., Bracke K.R., Joos G.F., Brusselle G.G. (2015). Transforming growth factor-beta superfamily in obstructive lung diseases. more suspects than TGF-beta alone. Am. J. Respir. Cell Mol. Biol..

[42] Li J., Panganiban R., Kho A.T., McGeachie M.J., Farnam L., Chase R.P., Weiss S.T., Lu Q., Tantisira K.G. (2020). Circulating MicroRNAs and Treatment Response in Childhood Asthma. Am. J. Respir. Crit. Care Med..

[43] Hayakawa K., Kawasaki M., Hirai T., Yoshida Y., Tsushima H., Fujishiro M., Ikeda K., Morimoto S., Takamori K., Sekigawa I. (2019). MicroRNA-766-3p Contributes to Anti-Inflammatory Responses through the Indirect Inhibition of NF-kappaB Signaling. Int. J. Mol. Sci..

[44] Cho W.C., Kwan C.K., Yau S., So P.P., Poon P.C., Au J.S. (2011). The role of inflammation in the pathogenesis of lung cancer. Expert Opin. Ther. Targets.

[45] Yin P., Peng R., Peng H., Yao L., Sun Y., Wen L., Wu T., Zhou J., Zhang Z. (2015). MiR-451 suppresses cell proliferation and metastasis in A549 lung cancer cells. Mol. Biotechnol..

[46] Pelaia C., Vatrella A., Gallelli L., Lombardo N., Sciacqua A., Savino R., Pelaia G. (2021). Role of p38 Mitogen-Activated Protein Kinase in Asthma and COPD: Pathogenic Aspects and Potential Targeted Therapies. Drug Des. Devel Ther..

[47] Lambrecht B.N., Hammad H., Fahy J.V. (2019). The Cytokines of Asthma. Immunity.

[48] Pelaia C., Vatrella A., Sciacqua A., Terracciano R., Pelaia G. (2020). Role of p38-mitogen-activated protein kinase in COPD: Pathobiological implications and therapeutic perspectives. Expert Rev. Respir. Med..

[49] DiMango E., Rogers L., Reibman J., Gerald L.B., Brown M., Sugar E.A., Henderson R., Holbrook J.T. (2018). Risk Factors for Asthma Exacerbation and Treatment Failure in Adults and Adolescents with Well-controlled Asthma during Continuation and Step-Down Therapy. Ann. Am. Thorac. Soc..

[50] Hu Y., Chen Z., Zeng J., Zheng S., Sun L., Zhu L., Liao W. (2020). Th17/Treg imbalance is associated with reduced indoleamine 2,3 dioxygenase activity in childhood allergic asthma. Allergy Asthma Clin. Immunol..

[51] Athari S.S. (2019). Targeting cell signaling in allergic asthma. Signal Transduct. Target. Ther..

[52] Liu Y., Miao Y., Gao X., Wang Y.Y., Wang H., Zheng Y.W., Zhao Z.Y. (2018). MicroRNA-200a Affects the Proliferation of Airway Smooth Muscle Cells and Airway Remodeling by Targeting FOXC1 via the PI3K/AKT Signaling Pathway in Ovalbumin-Induced Asthmatic Mice. Cell Physiol. Biochem..

[53] Bozinovski S., Vlahos R., Hansen M., Liu K., Anderson G.P. (2006). Akt in the pathogenesis of COPD. Int. J. Chronic Obs. Pulmon Dis..

[54] Wagner C., Uliczka K., Bossen J., Niu X., Fink C., Thiedmann M., Knop M., Vock C., Abdelsadik A., Zissler U.M. (2021). Constitutive immune activity promotes JNK- and FoxO-dependent remodeling of Drosophila airways. Cell Rep..

[55] Zaslona Z., Peters-Golden M. (2015). Prostanoids in Asthma and COPD: Actions, Dysregulation, and Therapeutic Opportunities. Chest.

[56] Bartel S., Schulz N., Alessandrini F., Schamberger A.C., Pagel P., Theis F.J., Milger K., Noessner E., Stick S.M., Kicic A. (2017). Pulmonary microRNA profiles identify involvement of Creb1 and Sec14l3 in bronchial epithelial changes in allergic asthma. Sci. Rep..

[57] Alashkar Alhamwe B., Miethe S., Pogge von Strandmann E., Potaczek D.P., Garn H. (2020). Epigenetic Regulation of Airway Epithelium Immune Functions in Asthma. Front. Immunol..

[58] Papi A., Bellettato C.M., Braccioni F., Romagnoli M., Casolari P., Caramori G., Fabbri L.M., Johnston S.L. (2006). Infections and airway inflammation in chronic obstructive pulmonary disease severe exacerbations. Am. J. Respir. Crit. Care Med..

[59] Roberts B.S., Hardigan A.A., Kirby M.K., Fitz-Gerald M.B., Wilcox C.M., Kimberly R.P., Myers R.M. (2015). Blocking of targeted microRNAs from next-generation sequencing libraries. Nucleic Acids Res..

