# Supplementary material for: Blood miRNAs Are Linked to Frequent Asthma Exacerbations in Childhood Asthma and Adult COPD
Source: Noncoding RNA. 2022 Apr 3;8(2):27. doi: 10.3390/ncrna8020027 (PMC9030787; doi:10.3390/ncrna8020027)
Supplement: Supplementary file 1 [file ncrna-08-00027-s001.zip › ncrna-1600832-supplementary.pdf]

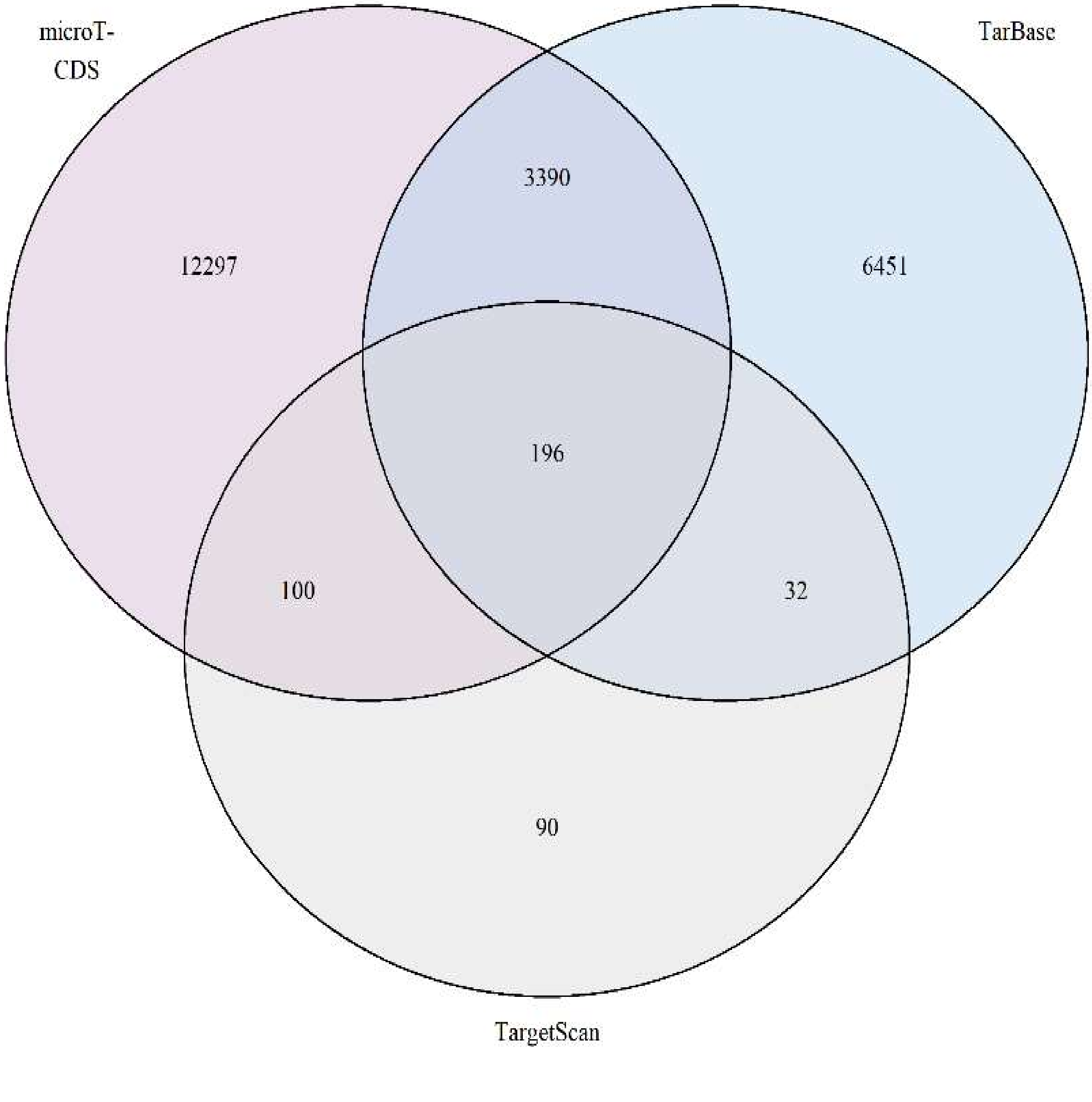

(A)

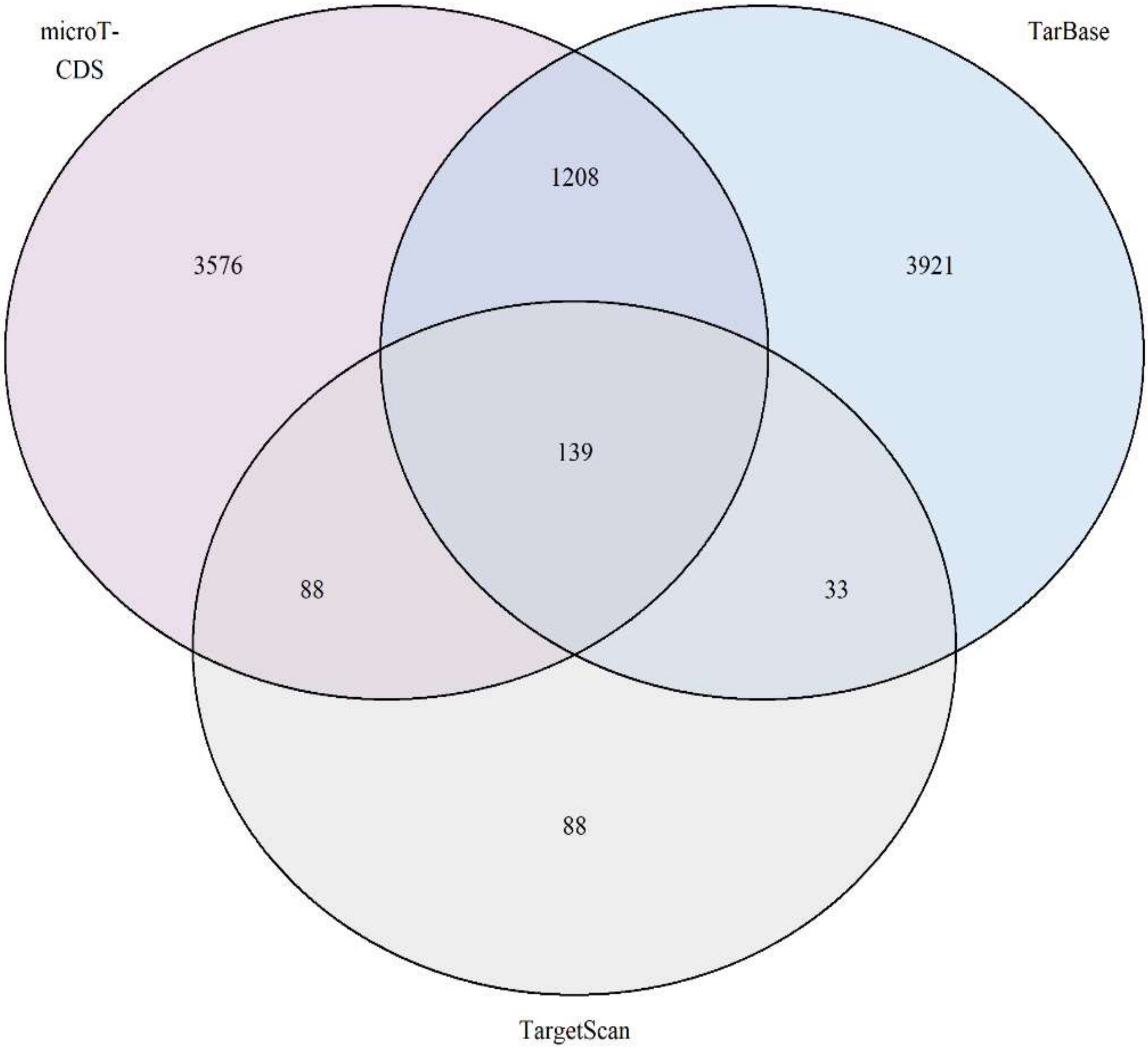

(B)

Supplemental Figure S1. Number of putative targets retrieved from microT-CDS, TarBase and TargetScan **(A)** for 20 DE miRNAs **(B)** 5 replicated miRNAs frequent and infrequent severe exacerbations.

Supplemental Table S1. List of all up- and down-regulated miRNAs between subjects without exacerbations and those experiencing severe exacerbators in COPDGene.

miR	log2FC	pvalue	FDR
hsa-miR-451b	-0.636	0.054	0.255
hsa-miR-7-5p	-0.524	0.064	0.255
hsa-miR-142-5p	-0.348	0.181	0.513
hsa-miR-6739-3p	-0.307	0.434	0.578
hsa-miR-29b-2-5p	-0.21	0.124	0.415
hsa-miR-500b-5p	-0.163	0.395	0.578
hsa-miR-6515-3p	-0.156	0.425	0.578
hsa-miR-500a-5p	-0.129	0.434	0.578
hsa-miR-93-3p	0.02	0.867	0.867
hsa-miR-664b-3p	0.031	0.831	0.867
hsa-miR-103a-2-5p	0.057	0.787	0.867
hsa-miR-331-3p	0.086	0.484	0.59
hsa-miR-4286	0.155	0.288	0.575
hsa-miR-550a-3p	0.164	0.361	0.578
hsa-miR-4433b-5p	0.168	0.501	0.59
hsa-miR-1296-5p	0.262	0.232	0.513
hsa-miR-642a-5p	0.273	0.231	0.513
hsa-miR-766-3p	0.289	0.036	0.241
hsa-miR-532-3p	0.311	0.022	0.208
hsa-miR-296-5p	0.391	0.021	0.208

Supplemental Table S2. List of 20 hub proteins from STRING.

Name	Degree	Betweenness Centrality	Closeness Centrality	Clustering Coefficient
UBA52	173	0.070	0.445	0.108
EGFR	159	0.057	0.451	0.099
MAPK1	133	0.045	0.442	0.102
JUN	114	0.030	0.430	0.122
ACTB	110	0.044	0.436	0.105
VEGFA	103	0.030	0.425	0.136
CDH1	102	0.030	0.427	0.116
HSPA8	102	0.026	0.426	0.136
MDM2	96	0.021	0.423	0.146
MTOR	90	0.020	0.425	0.168
SMARCA4	86	0.015	0.406	0.151
GSK3B	78	0.016	0.413	0.146
PIK3R1	74	0.016	0.400	0.178
FBXL19	72	0.009	0.377	0.351
EIF4E	69	0.012	0.403	0.169
H2AFV	68	0.014	0.381	0.146
HNRNPA1	68	0.012	0.389	0.146
CDK2	67	0.013	0.406	0.180
ATG7	66	0.008	0.397	0.393
RELA	66	0.008	0.407	0.169

Degree: *the number interactions (at the score threshold) of the protein.* ***Betweenness centrality:*** *proportion of shortest paths between all pairs of nodes which pass through the node.* ***Closeness centrality:*** *a measure of how short the shortest paths from a node to all other nodes are.* ***Clustering coefficient:*** *a measure of how connected a node is to other nodes in the network.*

COPDGene Phase 3

COPDGene® Investigators – Core Units

Administrative Center: James D. Crapo, MD (PI); Edwin K. Silverman, MD, PhD (PI); Barry J. Make, MD; Elizabeth A. Regan, MD, PhD

Genetic Analysis Center: Terri H. Beaty, PhD; Peter J. Castaldi, MD, MSc; Michael H. Cho, MD, MPH; Dawn L. DeMeo, MD, MPH; Adel El Boueiz, MD, MMSc; Marilyn G. Foreman, MD, MS; Auyon Ghosh, MD; Lystra P. Hayden, MD, MMSc; Craig P. Hersh, MD, MPH; Jacqueline Hetmanski, MS; Brian D. Hobbs, MD, MMSc; John E. Hokanson, MPH, PhD; Wonji Kim, PhD; Nan Laird, PhD; Christoph Lange, PhD; Sharon M. Lutz, PhD; Merry-Lynn McDonald, PhD; Dmitry Prokopenko, PhD; Matthew Moll, MD, MPH; Jarrett Morrow, PhD; Dandi Qiao, PhD; Elizabeth A. Regan, MD, PhD; Aabida Saferali, PhD; Phuwanat Sakornsakolpat, MD; Edwin K. Silverman, MD, PhD; Emily S. Wan, MD; Jeong Yun, MD, MPH

Imaging Center: Juan Pablo Centeno; Jean-Paul Charbonnier, PhD; Harvey O. Coxson, PhD; Craig J. Galban, PhD; MeiLan K. Han, MD, MS; Eric A. Hoffman, Stephen Humphries, PhD; Francine L. Jacobson, MD, MPH; Philip F. Judy, PhD; Ella A. Kazerooni, MD; Alex Kluiber; David A. Lynch, MB; Pietro Nardelli, PhD; John D. Newell, Jr., MD; Aleena Notary; Andrea Oh, MD; Elizabeth A. Regan, MD, PhD; James C. Ross, PhD; Raul San Jose Estepar, PhD; Joyce Schroeder, MD; Jered Sieren; Berend C. Stoel, PhD; Juerg Tschirren, PhD; Edwin Van Beek, MD, PhD; Bram van Ginneken, PhD; Eva van Rikxoort, PhD; Gonzalo Vegas Sanchez-Ferrero, PhD; Lucas Veitel; George R. Washko, MD; Carla G. Wilson, MS

PFT QA Center, Salt Lake City, UT: Robert Jensen, PhD

Data Coordinating Center and Biostatistics, National Jewish Health, Denver, CO: Douglas Everett, PhD; Jim Crooks, PhD; Katherine Pratte, PhD; Matt Strand, PhD; Carla G. Wilson, MS

Epidemiology Core, University of Colorado Anschutz Medical Campus, Aurora, CO: John E. Hokanson, MPH, PhD; Erin Austin, PhD; Gregory Kinney, MPH, PhD; Sharon M. Lutz, PhD; Kendra A. Young, PhD

Mortality Adjudication Core: Surya P. Bhatt, MD; Jessica Bon, MD; Alejandro A. Diaz, MD, MPH; MeiLan K. Han, MD, MS; Barry Make, MD; Susan Murray, ScD; Elizabeth Regan, MD; Xavier Soler, MD; Carla G. Wilson, MS

Biomarker Core: Russell P. Bowler, MD, PhD; Katerina Kechris, PhD; Farnoush Banaei-Kashani, PhD

COPDGene® Investigators – Clinical Centers

Ann Arbor VA: Jeffrey L. Curtis, MD; Perry G. Pernicano, MD

Baylor College of Medicine, Houston, TX: Nicola Hanania, MD, MS; Mustafa Atik, MD; Aladin Boriek, PhD; Kalpatha Guntupalli, MD; Elizabeth Guy, MD; Amit Parulekar, MD

Brigham and Women's Hospital, Boston, MA: Dawn L. DeMeo, MD, MPH; Craig Hersh, MD, MPH; Francine L. Jacobson, MD, MPH; George Washko, MD

Columbia University, New York, NY: R. Graham Barr, MD, DrPH; John Austin, MD; Belinda D'Souza, MD; Byron Thomashow, MD

Duke University Medical Center, Durham, NC: Neil MacIntyre, Jr., MD; H. Page McAdams, MD; Lacey Washington, MD

HealthPartners Research Institute, Minneapolis, MN: Charlene McEvoy, MD, MPH; Joseph Tashjian, MD

Johns Hopkins University, Baltimore, MD: Robert Wise, MD; Robert Brown, MD; Nadia N. Hansel, MD, MPH; Karen Horton, MD; Allison Lambert, MD, MHS; Nirupama Putcha, MD, MHS

Lundquist Institute for Biomedical Innovation at Harbor UCLA Medical Center, Torrance, CA: Richard Casaburi, PhD, MD; Alessandra Adami, PhD; Matthew Budoff, MD; Hans Fischer, MD; Janos Porszasz, MD, PhD; Harry Rossiter, PhD; William Stringer, MD

Michael E. DeBakey VAMC, Houston, TX: Amir Sharafkhaneh, MD, PhD; Charlie Lan, DO

Minneapolis VA: Christine Wendt, MD; Brian Bell, MD; Ken M. Kunisaki, MD, MS

Morehouse School of Medicine, Atlanta, GA: Eric L. Flenaugh, MD; Hirut Gebrekristos, PhD; Mario Ponce, MD; Silanath Terpenning, MD; Gloria Westney, MD, MS

National Jewish Health, Denver, CO: Russell Bowler, MD, PhD; David A. Lynch, MB

Reliant Medical Group, Worcester, MA: Richard Rosiello, MD; David Pace, MD

Temple University, Philadelphia, PA: Gerard Criner, MD; David Ciccolella, MD; Francis Cordova, MD; Chandra Dass, MD; Gilbert D'Alonzo, DO; Parag Desai, MD; Michael Jacobs, PharmD; Steven Kelsen, MD, PhD; Victor Kim, MD; A. James Mamary, MD; Nathaniel Marchetti, DO; Aditi Satti, MD; Kartik Shenoy, MD; Robert M. Steiner, MD; Alex Swift, MD; Irene Swift, MD; Maria Elena Vega-Sanchez, MD

University of Alabama, Birmingham, AL: Mark Dransfield, MD; William Bailey, MD; Surya P. Bhatt, MD; Anand Iyer, MD; Hrudaya Nath, MD; J. Michael Wells, MD

University of California, San Diego, CA: Douglas Conrad, MD; Xavier Soler, MD, PhD; Andrew Yen, MD

University of Iowa, Iowa City, IA: Alejandro P. Comellas, MD; Karin F. Hoth, PhD; John Newell, Jr., MD; Brad Thompson, MD

University of Michigan, Ann Arbor, MI: MeiLan K. Han, MD MS; Ella Kazerooni, MD MS; Wassim Labaki, MD MS; Craig Galban, PhD; Dharshan Vummidi, MD

University of Minnesota, Minneapolis, MN: Joanne Billings, MD; Abbie Begnaud, MD; Tadashi Allen, MD

University of Pittsburgh, Pittsburgh, PA: Frank Sciurba, MD; Jessica Bon, MD; Divay Chandra, MD, MSc; Joel Weissfeld, MD, MPH

University of Texas Health, San Antonio, San Antonio, TX: Antonio Anzueto, MD; Sandra Adams, MD; Diego Maselli-Caceres, MD; Mario E. Ruiz, MD; Harjinder Singh
